# Development of the Spittlebug *Mahanarva fimbriolata* under Varying Photophase Conditions

**DOI:** 10.1673/031.013.10501

**Published:** 2013-10-07

**Authors:** Amanda Daniela Simões, Eraldo Rodrigues Lima, Alexander Machado Auad, Tiago Teixeira Resende, Melissa Vieira Leite

**Affiliations:** 1 Entomology Department, Federal University of Viçosa, Viçosa-MG, Cep 36571-000, Brazil; 2 Embrapa Dairy Cattle Research Center, Cep 36038-330, Juiz de Fora - MG, Brazil

**Keywords:** biology, photoperiod, sugarcane

## Abstract

The effects of varying photophase conditions on biological parameters *of Mahanarva fimbriolata* (Stal) (Hemiptera: Cercopidae), nymphs and adults were evaluated. Eggs of a late developmental stage were placed near sugarcane roots (cultivar RB739735) maintained in a greenhouse (21 ± 7° C, 90 ± 10% RH). Nymphs and adults were exposed to the following photophase conditions: a) 13:11 L:D as nymphs and adults, b) 13:11 as nymphs and 12:12 as adults, c) 12:12 as nymphs and adults, and d) 12:12 as nymphs and 13:11 as adults. Exposure of nymphs to 13 hr of light significantly reduced the duration of the nymphal stage and the number of nymphs that matured to adults. The duration of the nymphal stage was longer in individuals developing into females than in those developing into males. There was an increase in the longevity of adults kept at 13 hr of light since the nymphal stage. The average longevity of adult males and females was approximately the same. The sex ratio was similar under all photophase conditions. The life cycle of insects kept at 12 and 13 hr of light during nymphal and adult stages respectively was extended. The total life cycle was significantly longer in *M. fimbriolata* females than males. The different photophase conditions did not affect the reproductive potential of *M. fimbriolata*. Females produced more diapausing than non-diapausing eggs, except when under 13- and 12-hr light conditions. There was no significant difference in the number of diapausing and non-diapausing eggs produced by females under the other photophase conditions.

## Introduction

Spittlebugs occur in various parts of the world, but are most common in tropical and subtropical regions (Fewkes 1969). Species in the genus *Mahanarva* occur in Costa Rica, Panama, and throughout South America. *Mahanarva* contains 32 species, some of which are pests of grasses or sugarcane (Peck 2004). Nymphs of *Mahanarva fimbriolata* (Stal) (Hemiptera: Cercopidae) cause physiological disorders in their host plants by piercing plant tissues with their mouthparts, which reach vessels of the woody root, xylem, and phloem, deteriorating these vessels and thus preventing the flow of water and nutrients throughout the plant (Garcia et al. 2006). The feeding behavior of adults causes the symptom known as “burning of sugarcane” as a result of toxins being injected into plants tissues, causing a reduction in the size and thickness of the in-ternodes (Guagliumi 1972, 1973).

Climatic factors such as temperature, rainfall, high humidity, and photoperiod during spring and summer seasons in tropical regions influence the duration of the life cycle, number of population peaks, and consequently, the number of spittlebug generations per year (Mendonca 1996). During autumn and winter, seasons when climatic conditions are unfavorable for development of *M. fimbriolata*, there is a significant reduction in nymphs and adults in the field because eggs increasingly enter a state of diapause.

Climatic factors can greatly influence the population dynamics of *M. fimbriolata*. For example, daily changes of 10 or 15 min in day length (photophase) can cause significant reductions or accelerations in growth, maturation, and oviposition rates (Denlinger 2002). In addition, adding 1 hr of light to the photophase can convert individuals from onepattern of development to another (Nishizuka et al. 1998; Hastings 2001). Based on this assumption, it was hypothesized that *M. fimbriolata* maintained with a photoperiod of 13:11 L:D (nymphal stage) and 13:11 (adult stage) and 13:11 (nymphal stage) and 12:12 (adult stage) would give rise to significantly higher densities of nymphs and adults. It was also hypothesized that maintaining *M. fimbriolata* with a photoperiod of 12:12 L:D (nymphal stage) and 12:12 (adult stage) and 12:12 (nymphal stage) and 13:11 (adult stage) would result in a significant reduction of nymphs and adults and an increase in diapaus-ing eggs. This study assessed the influence of 12:12 L:D and 13:11 photoperiods, constant or alternated, between nymphal and adult stages of *M. fimbriolata* on the duration of these stages and life cycle (nymphal plus adult stages), sex ratio, reproduction, and production of diapausing, non-diapausing, and nonviable eggs.

**Table 1.**
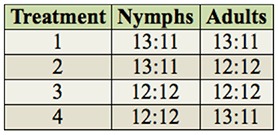
Different photoperiods (L:D) to which *Mahanarva fimbriolata* nymphs and adults were subjected.

## Materials and Methods

Approximately 1,000 *M. fimbriolata* adults were captured using standard entomology nets during two consecutive days of collection in sugarcane plots (cultivar RB739735) at the Coronel Pacheco Experimental Field of the Embrapa Dairy Cattle Research Center (www.embrapa.br/ ) during the second fortnight of September 2008. Later, the specimens were transferred to the laboratory, and males and females were separated. Fifty couples were released inside transparent acrylic cages (30 × 30 × 60 cm), each containing 4 sugarcane plants (60 days old, cv. RB73-9735), maintained in PVC containers (10 cm high × 7.5 cm in diameter) in the laboratory. A cover was placed on top of the container, under which a section of gauze moistened with distilled water was kept, which served as an oviposition substrate. For the removal of eggs deposited on the substrate, the gauze was placed on a set of sieves and subjected to water jets so that the eggs were held at the thinnest sieve (400-µm mesh size). Eggs were placed in petri dishes (2 cm high × 5 cm in diameter) lined with moistened filter paper and kept in a climatic chamber at 28° C, 70 ± 10% RH, and a 14:10 L:D photoperiod. The filter paper was moistened daily, and embryonic development was observed up to the stage E4, close to hatching. This stage is characterized as the one in which eggs have two red spots on each side of the operculum, corresponding to the eyes, and larger red spots representing the nymphas abdominal pigments (Peck 2002). These eggs were used in the tests to obtain nymphs and adults.

To ensure uniformity of treatments within the greenhouse, 100-W lamps remained on daily for 13 hr. Cages were covered with white voile fabric. In order to simulate dark periods, black voile fabric covered the white caps in the periods corresponding to the scotophase of each treatment. The average reduction of light incidence within each experimental unit during the light regimes (white cloths) and scotophase (white cloths covered by black cloths) was 15% and 65%, respectively, compared to the light incidence inside the greenhouse. The incident light was recorded with a Decagon LP80 Canopy Analyzer (www.decagon.com ).

Photophase conditions used during immature and adult stages of *M. fimbriolata* were selected from the average light incidence in months during which a greater number of normal, non-diapausing eggs were found (i.e., from September to March: 13 hr) or in months during which a greater number of diapausing eggs were found (i.e., from April to August: 12 hr). Photoperiod records were obtained through the Observatiorio Nacional (2008) based in Coronel Pacheco, Minas Gerais, Brazil, which was also the sampling site for *M. fimbriolata* adults used in this test.

For tests with nymphs, eggs were obtained at stage E4 (from a breeding colony), and 50 eggs were placed on each 60-day-old sugarcane plant (cv. RB73-9735) potted within cages with metal frames (70 cm × 40 cm × 40 cm) and covered with voile fabric to simulate the photoperiods so that nymphs were subjected to 12:12 L:D or 13:11. Biological parameters relating to the duration of the nymphal stage, regardless of sex, and nymphs that developed into adults were evaluated.

For tests with adults, 5 *M. fimbriolata* adult couples (newly emerged) were placed in clear plastic cylindrical cages (50 cm high × 10 cm in diameter) containing a 60-day-old sugarcane plant (cv. RB73-9735). A section of gauze moistened with distilled water encircled the base of each cage to serve as an oviposition substrate. These breeding units were kept in cages of steel covered with voile (as described above) to simulate photoperiods. The experimental treatments were: adults from nymphs maintained at 13:11 L:D were subjected to a photoperiod of 13:11 (treatment 1) or 12:12 (treatment 2), and adults from nymphs maintained at 12:12 L:D were subjected to photoperioids of 12:12 (treatment 3) or 13:11 (treatment 4) (Table 1). The longevity of all adults, longevity of males and females, total life cycle duration, total life cycle duration of males and females, and the sex ratio were evaluated. Eggs from each treatment were placed in Petri dishes lined with filter paper moistened with distilled water and kept in climate chambers at 28° C, 70 ± 10% RH, and the same photophase conditions as those of the adults. The egg viability was checked daily. Eggs were classified according to the criteria adopted by Castro et al. (2005), in which nymphs whose eggs hatch during the first 30 days are non-diapausing and those that hatch after this period are diapausing. The number of normal, diapausing, and non-viable eggs, and the total average number of eggs laid per female, were recorded each day. Temperature and RH were recorded using a portable thermo-hygrograph kept inside the climate chambers; the average values obtained were 21 ± 7° C and 90 ± 10% RH.

**Figure 1.**
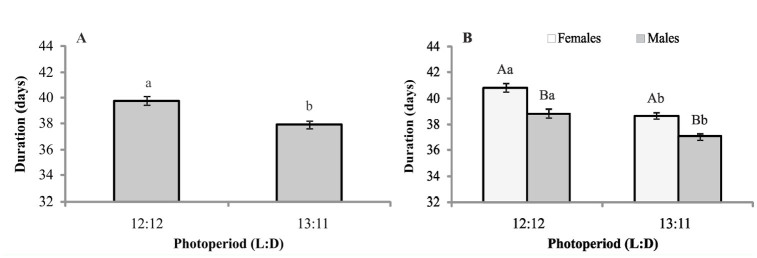
Average number of days ± SE in the nymphal stage, regardless of sex (A) and nymphal stage of *Mahanarva fimbriolata* originating from adult females and males (B) in sugarcane and subjected to different photoperiods. Means followed by the same capital letter within treatments and lower case letter among treatments do not differ significantly (Scott and Knott test, *p* < 0.05). High quality figures are available online.

**Figure 2.**
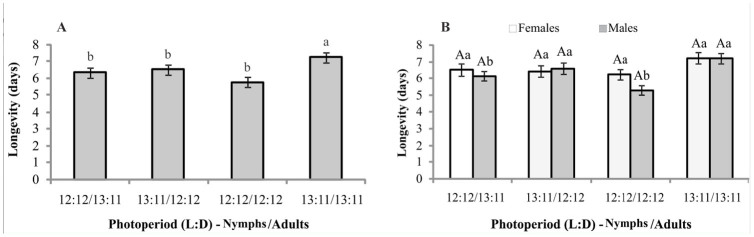
Longevity of *Mahanarva fimbriolata* (in mean number of days ± SE) of the adult stage regardless of sex (A) and with respect to sex (B). Nymphs and adults were exposed to different photoperiods. Means followed by the same capital letter within treatments and lower case letter among treatments did not differ significantly (Scott and Knott test). High quality figures are available online.

A randomized block design in a factorial arrangement with 20 replications of two photophases (12:12 hr and 13:11 hr) for the nymphal stage, and depending on the photophase conditions, 10 replications for the adult stage, was used. The ANOVA and the means compared by the Scott and Knott test at a 5% significance level were used.

## Results

The duration of the nymphal stage was significantly shorter (F = 24.94, df = 1, *p* < 0.0001) for nymphs reared with a photoperiod of 13:11 L:D compared to nymphs reared with a photoperiod of 12:12 (Figure 1A). The same result was verified when separately analyzing the nymphs developing into females (F = 17.72, df = 1, *p* = 0.0001) and males (F = 12.32, df = 1, *p* = 0.0009) (Figure 1B). The duration of the nymphal stage of insects giving rise to females was longer than that resulting in males when the immature insects were maintained with a photoperiod of both 12:12 L:D(F= 15.07, df= 1, *p* = 0.0003) and 13:11 (F = 10.12, df = 1, *p* = 0.0024) (Figure 1B).

**Figure 3.**
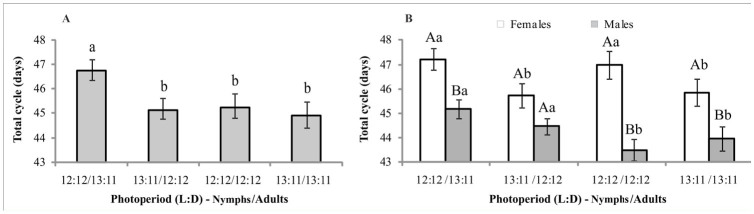
Life cycle (nymphal stage and adult longevity) in mean number of days ± SE regardless of sex (A) and total cycle of females and males (B) of *Mahanarva fimbriolata* in sugarcane. Nymphs and adults were exposed to different photoperiods. Means followed by the same capital letter within treatments and lower case letter among treatments do not differ significantly (Scott and Knott test). High quality figures are available online.

In regards to the adult stage of *M. fimbriolata, *it was found that a 13:11 L:D photoperiod beginning at nymph outbreak caused significantly higher changes in adult longevity (F = 3.60, df = 3, *p* = 0.0183). In treatments with alternating photoperiods, depending on the development stage of the insect and the treatment in which the photoperiod was 12:12 L:D for both stages, longevities were significantly equal (Figure 2A). Regardless of the photoperiod, the average longevity between males and females was significantly equal. Among photoperiods, the same duration of the adult stage in females was found (F = 0.93, df = 3, *p =* 0.432); however, males kept in photoperiods of 12:12 L:D (nymphal stage) and 13:11 (adult stage) and 12:12 (both nymphal and adult stages) lived for significantly shorter periods (F = 3.262, df = 3,*p* = 0.0268) (Figure 2B). Concerning the sex ratio, it was similar in the different photophase conditions and ranged from 0.39 to 0.48.

Considering the total life cycle (nymph and adult) in the treatments where the photoperiod was alternated depending on the stage of insect development, nymphs kept at a lower light incidence (12:12 L:D as nymphs, 13:11 as adults) showed an extension of the life cycle (F = 4.06, df = 3, *p* = 0.0105). No significant difference was observed between the treatments that had constant photoperiods (12:12 L:D both as nymphs and adults and 13:11 both as nymphs and adults) and those that had a higher light incidence for nymphs (13:11 L:D for nymphs, 12:12 for adults) (Figure 3A).

The total life cycle of individuals that developed into females was longer than the total life cycle of individuals that developed into males in the photoperiod conditions of 12:12 L:D (nymphs) and 13:11 (adults), 12:12 (nymphs) and 12:12 (adults), and 13:11 (nymphs) and 13:11 (adults). Among photoperiod conditions evaluated, females lived significantly (F = 2.54, df = 3, *p* = 0.0637) longer when they were maintained in the nymphal stage with a photoperiod of 12:12 L:D (Figure 3B). In those treatments where photoperiod conditions were alternated depending on the stage of insect development, males had high mean values (F = 2.78, df = 3, *p* = 0.0479) (Figure 3B).

The different photoperiods did not affect the average fertility (8.18–14.19 eggs per female), number of eggs per female per day (1.37–2.31), or the total average number of eggs produced by *M. fimbriolata* (42.20–65.60).

**Figure 4.**
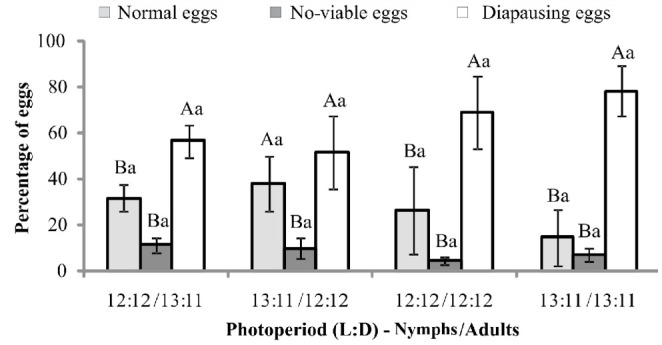
The average number of normal, diapausing, and nonviable eggs produced by *Mahanarva fimbriolata* in sugarcane. Nymphs and adults were exposed to different photoperiods. Means followed by the same capital letter within treatments and lower case letter among treatments do not differ significantly (Scott and Knott test). High quality figures are available online.

Regarding the classification of eggs, except for insects kept with a photoperiod 13:11 L:D (nymphs) and 12:12 (adults) light regime, where the average number of normal and diapausing eggs were statistically equal, the number of diapausing eggs produced was always higher than that of normal ones. Among the different photophase conditions, there was no significant difference in the number of normal (F = 1,51, df = 3, *p* = 0.2156) and diapausing (F = 2.17, df = 3, *p* = 0.0957) eggs (Figure 4).

## Discussion

Factors such as temperature, availability of a suitable food source, and day length influence the development of the nymphal stage in spit-tlebugs. In addition, the interaction of photoperiod and temperature is important in enabling spittlebug development. According to Musolin and Ito (2008), nymphs of the *Onus* spp. developed at a significantly slower rate under 14 hr of light but at a faster rate at 10 and 16 hr of light at 20° C. However, under the same photoperiod conditions and higher temperatures (24° C and 28° C), the period of development reduced with increasing light incidence. Similarly, in our study, a 13-hr photophase reduced the nymphal stage of *M*.*fimbriolata*. In contrast, Garcia (2006) found no significant differences in development time when nymphs of the same species were under 10-, 12-, and 14-hr photoperiods.

The results obtained for the average duration of the nymphal stage of *M. fimbriolata* in different light regimes (12 or 13 hr) in our study were similar to those reported by Garcia et al. (2006) for the same species of spittlebug undergoing a 14-hr photoperiod. However, Garcia (2006) found lower values when nymphs were in 10-, 12-, and 14-hr photophase conditions.

Previous studies showed a variation in the length of the nymphal period in different light regimes for cercopids of the genus *Mahanarva*. Under laboratory conditions using a 12-hr photophase, Souza (1967) recorded a nymphal period of 60–70 days for *Mahanarva posticata*. Guagliumi (1969), using a 12-hr light regime, recorded a nymphal period varying from 36 to 90 days. Ribemboim and Cisneiros (1967) found that *Mahanarva indicata* had a nymphal period varying from 43 to 74 days under a 14-hr light regime. However, Rodriguez and Peck (2007) found an average nymphal period of 48.4 days for *Mahanarva andigena* under uncontrolled photoperiod conditions. Thus, there is high inter- and intra-specific variation in the duration of the nymphal stage of *Mahanarva*, even in similar photophase conditions. The observation that the nymphal stage of males was shorter than that of females in *M. fimbriolata* justifies the occurrence of males prior to females in population surveys of this pest for four consecutive years in Coronel Pacheco, MG, Brazil (A. M. Auad, personal observation). This finding coincides with those of Sotelo (2004), who recorded the emergence of males of *Aeneolamia varia* before females. According to Musolin and Ito (2008), the early emergence of males is ecologically important because it ensures that males are ready to mate with females as soon as they emerge.

The duration of the nymphal stage of *M. fimbriolata* that originated from males or females was lower at a higher light incidence (13 hr). This response may be variable depending on the species, geographic region, and season. Musolin and Ito (2008) found a trigger signal to accelerate growth of *Orius* spp. nymphs in temperate regions subjected to reduced light incidence at the end of autumn. Moreover, early emergence allows more time for mating in autumn and accumulating fat reserves necessary for successful overwintering of females. The range of time required for the nymphal stage of *M. fimbriolata* originating from males or females in this study is similar to that found for the same species by Garcia et al. (2006) and Garcia (2006) under similar photophase conditions.

The longevity of *M. fimbriolata* decreased when maintained, at least during one of the stages, at a lower light regime. Garcia (2006) also observed a progressive reduction in the longevity of *M. fimbriolata* adults, regardless of sex, maintained at a 10-, 12-, or 14-hr photophase due to an increase in light exposure. Denlinger (2002) found that a small change of 10 or 15 min of daylight caused a significant change in the longevity of some species. On similar lines, Nishizuka et al. (1998) and Hastings (2001) found that a change of 1 hr of daylight modified the developmental state of individuals due to a change in photoperiod.

Contrary to what was observed during the immature stage in our study, photoperiod had no effect on the longevity between males and females. The interaction of the shortest day lengths during the nymphal stage reduced male longevity compared with those kept under the longest light regime in the immature stage. For females, light regimes had no effect on longevity. Similarly, Garcia (2006) found no significant difference in this parameter for both sexes in 10-, 12-, and 14-hr photoperi-ods, despite having observed a higher average longevity than that in our study. Grisoto (2008) also reported no differences in longevity between males and females during a 14-hr photophase. However, Garcia et al. (2006) reported significant differences in longevity between males and females in a 14-hr photophase.

Garcia et al. (2006) found adult longevities that were 3 times higher than those reported in our study for the same cercopid species under similar climatic conditions. This difference may be due to the plant species offered as food for insects, as evident in the work of Grisoto (2008), wherein the longevity was 2.5 times higher for males fed on sugarcane culti-var SP 80-1842 compared to those fed on *Brachiaria brizantha* cultivar IAC-BBS8.

The sex ratio of *M. fimbriolata* was similar to that described by Garcia et al. (2006), who found a 0.50 sex ratio in three successive generations when insects were under a 14-hr photophase. Grisoto (2008) found that sex ratio ranged from 0.35 to 0.76 in *M. fimbriolata*, although plant species had no effect on sex ratio.

When the photophase was alternated and exposed nymphs to a lower light incidence, the life cycle (nymph and adult) of *M. fimbriolata* increased (Figure 3A). Because insects remain for a longer time in the nymphal stage (40 days) than in the adult stage (about 6 days), it was speculated that the immature stage has a greater influence on the total duration of the life cycle. The observation that the total life cycle of individuals originating from *M. fimbriolata* females was higher than those originating from males (Figure 3B) could be due mainly to the duration of the nymphal stage, because longevity did not differ significantly between sexes (Figure 2B). The values observed for the entire duration of the life cycle were similar to those observed by Garcia (2006) and Grisoto (2008) in similar pho-tophase conditions. However, Garcia et al. (2006) found lower values for the duration of the life cycle for *M. fimbriolata* on the same host plant under a 14-hr photophase, as did Ballesteros and Galego (1999) studying *Ma-hanarva* spp. on *Brachiaria decumbens*.

The photophase conditions in our study did not alter the average fertility of *M. fimbriolata*, which was in concordance with the results of Garcia (2006) for the same species. However, the values recorded for the number of eggs laid in our study were lower than those obtained by Garcia (2006) and Garcia et al. (2006), which reported a fertility value between 140 and 342 eggs per female, respectively, in a 14-hr photophase. Whereas the conditions of photophase and humidity were similar to those used in our study, it is believed that these variations were promoted by temperature, which oscillated during our study and was constant in the research of Garcia (2006) and Garcia et al. (2006). Another factor is related to the plant genotype, which interferes with spittlebugs' fertility. Grisoto (2008) found 21-187 eggs per female, depending on the grass offered to adults of *M. fimbriolata* kept under identical conditions to those of Garcia (2006) and Garcia et al. (2006).

Light conditions did not affect the number of eggs laid by *M. fimbriolata* females each day. Average fecundity divided by longevity of females kept at 25° C and a 14-hr photophase, according to data recorded in the research ofGarcia (2006) and Garcia et al. (2006), were on average 16 and 22 times higher than in our study. However, such surveys were conducted under different conditions of temperature and host plant. The average number of non-viable eggs in all treatments was similar to the number found by Garcia (2006), who found 8.5% and 9% non-viable eggs of *M. fimbriolata* under light regimes of 10 and 12 hr, respectively. This result does not corroborate with the results of Magalhaes et al. (1987), who found just 36.5% viable eggs for *Deois incompleta* in the laboratory. This difference may be due to intrinsic features of each species.

Hairson Jr. and Kearns (1995) found that photophase regulates diapause in several species in 13 insect orders. Saunders (1965, 1997) and Saunders et al. (1988, 1997, 2003, 2004) described the mechanism involved in regulation. The average number of diapausing eggs observed in all treatments was similar, disagreeing with the results obtained by Evans (1972) in studies on *Aeneolamia varia* in sugarcane. He found that most diapausing eggs in treatments that had a photophase between 12.18 and 12.38 hr differed from those kept in alternate photophases of 10 and 14 hr. This variation can also occur in other insects, such as the pink bollworm, *Pectinophora gossy-piella*, which is capable of distinguishing between 12 hr of light (98% diapause) and 14 hr of light (5% diapause) per day in the immature stage (Pittendrigh et al. 1970). Although photoperiod is the environmental factor that stimulates the onset of diapause, temperature can modify this response (Tauber 1986). Wang et al. (2009) demonstrated that high temperature (35° C) reversed the effect of short days on diapause induction in *Sericinus montelus* pupae. As in the our study, the insects were in a greenhouse at an average temperature of 21° C, but the temperature exceeded 35° C at certain periods of the day, which may have inhibited the occurrence of diapausing eggs even when insects were exposed to low light incidence. It is important to emphasize that the production of diapausing eggs is an important strategy for the persistence of spittlebugs as a pest in sugar cane. However, photophase conditions did not trigger an increase in the number of diapausing eggs in this study.

In future studies, researchers should test other light incidences in association with different temperatures to predict the occurrence of peaks in the abundance of spittlebugs and availability of specimens for experimental breeding. This information may contribute to the integrated management of these cercopids by assisting in decision making on the control method to be adopted.
